# Polysaccharopeptide enhanced the anti-cancer effect of gamma-tocotrienol through activation of AMPK

**DOI:** 10.1186/1472-6882-14-303

**Published:** 2014-08-16

**Authors:** Ji Liu, Eunice Yuen-Ting Lau, Jiezhong Chen, Joan Yong, Kai Dun Tang, Jessica Lo, Irene Oi-Lin Ng, Terence Kin-Wah Lee, Ming-Tat Ling

**Affiliations:** Australian Prostate Cancer Research Centre-Queensland & Institute of Health and Biomedical Innovation, Queensland University of Technology, Kragujevac, Qld Australia; Department of Pathology, Faculty of Medicine, the University of Hong Kong, Hong Kong, SAR, China; School of Biomedical Sciences, the University of Queensland, Kragujevac, Qld Australia; Translational Research Institute, 37 Kent Street, Brisbane, QLD 4102 Australia; Room 704, 7/F, Faculty of Medicine Building, 21 Sassoon Road, Hong Kong, SAR, China

**Keywords:** Polysaccharopeptide, Tocotrienol, AMPK, Prostate cancer

## Abstract

**Background:**

Prostate cancer (PCa) frequently relapses after hormone ablation therapy. Unfortunately, once progressed to the castration resistant stage, the disease is regarded as incurable as prostate cancer cells are highly resistant to conventional chemotherapy.

**Method:**

We recently reported that the two natural compounds polysaccharopeptide (PSP) and Gamma-tocotrienols (γ-T3) possessed potent anti-cancer activities through targeting of CSCs. In the present study, using both prostate cancer cell line and xenograft models, we seek to investigate the therapeutic potential of combining γ-T3 and PSP in the treatment of prostate cancer.

**Result:**

We showed that in the presence of PSP, γ-T3 treatment induce a drastic activation of AMP-activated protein kinase (AMPK). This was accompanied with inactivation of acetyl-CoA carboxylase (ACC), as evidenced by the increased phosphorylation levels at Ser 79. In addition, PSP treatment also sensitized cancer cells toward γ-T3-induced cytotoxicity. Furthermore, we demonstrated for the first time that combination of PSP and γ-T3 treaments significantly reduced the growth of prostate tumor *in vivo*.

**Conclusion:**

Our results indicate that PSP and γ-T3 treaments may have synergistic anti-cancer effect *in vitro* and *in vivo*, which warrants further investigation as a potential combination therapy for the treatment of cancer.

## Background

Prostate cancer (PCa) is the most common type of solid tumor in men around the world and is a leading cause of morbidity and mortality. Due to the slow growing nature of the tumor, many prostate cancer patients have already developed metastatic disease, where surgery is no longer feasible. The only frontline treatment available for the PCa patient at the advanced stage is hormone ablation therapy. Unfortunately, the majority of patients will eventually relapse and develop castration-resistant prostate cancer (CRPC), a fatal and terminal stag. There is currently no curative treatment against hormone refractory prostate cancer (HRPC) since the tumor frequently develops resistance to conventional chemotherapy, with the most effective treatment (Docetaxel, a microtubule-disrupting agent) extend patient survival for an average of only two months and is associated with significant side effects [1]. Thus, there is an urgent need for a better therapy for CRPC that shows improved treatment efficiency and minimal side effects.

We and other have reported that a number of bioactive food compounds have potent anti-cancer effect, particularly against the cancer stem cell (CSC) population, which has been suggested to play a key role in the development and progression of prostate cancer. For example, we previously demonstrated that gamma-tocotrienol (γ-T3) extracted from palm oil suppresses prostasphere formation and tumor development of prostate cancer cells [2]. In addition, a triterpene extracted from fruits was also found to target liver CSCs and sensitize cells to cisplatin treatment [3]. More recently, we have shown that polysaccharopeptide (PSP) isolated from Turkey tail (known as *Coriolus versicolor* or Yun-zhi) also inhibit prostate CSC self-renewal *in vitro* and prevent prostate cancer development *in vivo*[4]. These findings demonstrated the potential of natural compounds as effective anti-cancer agent through targeting of CSCs.

Although natural compounds such as γ-T3 or PSP both demonstrated potent anti-cancer effect, they were found to act through different downstream mechanisms. For example, γ-T3 has been shown to inhibit prostate cancer cell invasion by suppressing the epithelial to mesenchymal transition [5]. It was also found to regulate a number of key prosurvival signaling pathways, such as NF-KappaB [6] and PI3K [7], and as a result induce apoptosis of the cancer cells. PSP, on the other hand, was found to inhibit CSC self-renewal without affecting the survival of the cancer cells. In particular, oral intake of PSP was found to have immunomodulatory effect in breast cancer patients, as evidenced by the significant induction of the T-helper lymphocytes and the B-lymphocytes [8]. It is thus tempting to speculate that combination of these compounds may achieve a synergistic anti-cancer effect through targeting of multiple signalling pathways.

Here, we investigated the *in vitro* and *in vivo* anti-cancer effect of combining PSP and γ-T3. We found that the PSP significantly enhanced the cytotoxicity effect of γ-T3 on cancer cells, which was associated with activation of AMPK. Meanwhile, treatment of the cells with PSP and γ-T3 also lead to inactivation of the acetyl-CoA carboxylase (ACC). Furthermore, oral supplementation of a combined dosage of PSP and γ-T3 was found to have a more potent anti-tumor effect than either agent alone. Our findings highlight the potential of combining PSP with γ-T3 in the treatment of cancer.

## Methods

### Polysaccharopeptide (PSP) and gamma-tocotrienol (γ-T3)

PSP extracted from Yun-zhi was kindly provided by Wonder Herb Health Products, Ltd. The PSP powder was dissolved in autoclaved Milli Q water at a concentration of 30 mg/ml by mixing in a rotator at room temperature overnight. The PSP solution was stored at -20°C. For cell culture study, PSP stock was sterilized with 0.2 μm filtration prior to use. In the animal study, PSP was fed directly to mice. γ-T3 was provided by Davos Life Science Pte. Ltd, Singapore and was dissolved in absolute ethanol (2.5 μg/ml, 3.5 μg/ml and 5 μg/ml) and stored at -20°C. Using the corresponding T3 isomers as reference standards, the purity of γ-T3 was verified to be ≥97% by high performance liquid chromatography (HPLC).

### Cell lines and culture conditions

Prostate cancer cell lines PC3 (ATCC, Rockville, MD) were maintained in RPMI 1640 medium (Invitrogen, Carlsbad, CA) supplemented with 1% (w/v) penicillin-streptomycin (Invitrogen, Carlsbad, CA) and 5% fetal bovine serum (Invitrogen, Carlsbad, CA). All cell types were kept at 37°C in a 5% CO_2_ environment.

### Generation of PC-3 cells stably expressing GFP

A GFP-expressing PC-3 cell line, PC3-GFP, was generated using the Viralpower Lentiviral gene expression system (Invitrogen, Carlsbad, CA) according to the manufacturer’s instructions. Briefly, supernatant containing the lentivirus was mixed with polybrene (8 μg/ml) and used to infect PC-3 cells. After infection, positive transfectants were selected as a pool by treatment with blasticidine (10 μg/ml) for six days.

### Western blotting

Detailed experimental procedures have been described previously [4]. Briefly, whole cell lysates were prepared by resuspending cell pellets in cell lysis buffer (20 mM Tris-HCl, 150 mM NaCl, 1 mM Na_2_EDTA, 1 mM EGTA, 1% Triton, 2.5 mM sodium pyrophosphate, 1 mM beta-glycerophosphate, 1 mM Na_3_VO_4_, 1 μg/ml leupeptin). Protein concentration was determined using Pierce BCA Protein Assay Kit (Pierce, Rockford, IL). Protein extract was loaded onto an SDS-polyacrylamide gel, separated by electrophoresis and then transferred to a PVDF membrane (Millipore, Billerica, MA). The membrane was then incubated with primary antibodies against AMPKα, phospho-AMPKα (Thr172) and phospho-ACC (Ser79) (Cell signaling, Technology Inc, Beverly, MA) and γ-tubulin (Sigma-Aldrich, St Louis, MO) overnight at 4°C. After washing with TBS-T, the membrane was incubated with rabbit IgG secondary antibodies, and the signals were visualized using the ECL western blotting system (Millipore, Billerica, MA).

### Colony formation assay

The colony formation assay was carried out as described previously [2]. Briefly, PC3 cells were seeded 12-well plate (100 cells per well). Cells were cultured for 14 days in the presence of γ-T3 at 2.5 μg/mL, 3.5 μg/mL or 5 μg/mL and/or PSP at 30 μg/mL. At the end of the experiment, the number of the colonies formed was counted. Colony forming ability was normalized as a percentage of untreated cells. Each experiment was repeated in triplicate, and each data point represents the mean and standard derivation. Statistical difference was determined by Student *t*-test and was considered as significance if p < 0.05.

### *In vivo*treatment of PSP and γ-T3

The xenograft was established in 4- to 6-week-old male athymic nude mice (BALB/c-*nu/nu*) with GFP-labeled PC3 prostate cancer cell line (PC3-GFP). Treatment was started once the size of the xenograft reached approximately 4 × 4 mm^2^ (length × width) in size after subcutaneous injection of 1 × 10^6^ of PC3-GFP cells, during which the mice were randomly assigned into four groups and each group consisted of 5 mice: 1) γ-T3 (50 mg/Kg/day), 2) PSP (300 mg/Kg/day), and 3) γ-T3 (50 mg/Kg/day) plus PSP (100 mg/Kg/day) and 4) DMSO and PBS as a control. γ-T3 and PSP were administered to the mice intraperitoneally and orally respectively. After 30 days of various treatments, the effects on tumor growth were measured and recorded with a Maestro imaging system (CRI Inc. Woburn, MA). Statistical difference was determined by a two-tailed *t*-test and was considered significant if p < 0.05. All the animal experiments have been approved by the animal ethics committee of The University of Hong Kong and the surgical and animal handling procedures were carried out according to the guidelines of the Committee on the Use of Live Animals in Teaching and Research (CULATR), The University of Hong Kong.

### Immunostaining

Tissues were formalin fixed and paraffin embedded. Four-micrometer sections were cut, dewaxed in xylene and graded alcohols, hydrated, and washed in PBS. After pretreatment in a microwave oven (12 minutes in sodium citrate buffer [pH 6]), the endogenous peroxidase was inhibited by 0.3% H_2_O_2_ for 30 minutes, and the sections were incubated with 10% normal goat serum for 30 minutes. Rabbit polyclonal anti–phospho-AMPKα (Thr172) (1:100) and anti-phospho-ACC (Ser79) (Cell Signal Technology) was applied overnight in a humidity chamber at 4°C. (A standard avidin-biotin peroxidase technique (Dako) was applied. Briefly, biotinylated goat anti-mouse immunoglobulin and avidin-biotin peroxidase complex were applied for 30 minute each, with 15-minute washes in PBS. The reaction was finally developed with the Dako Liquid DAB + Substrate Chromogen System (Dako). Slides were imaged on an Aperio Scanscope CS imager, generating 0.43-μm/pixel whole slide images.

## Results

### PSP enhances the cytotoxic effect of γ-T3

**Figure 1:**
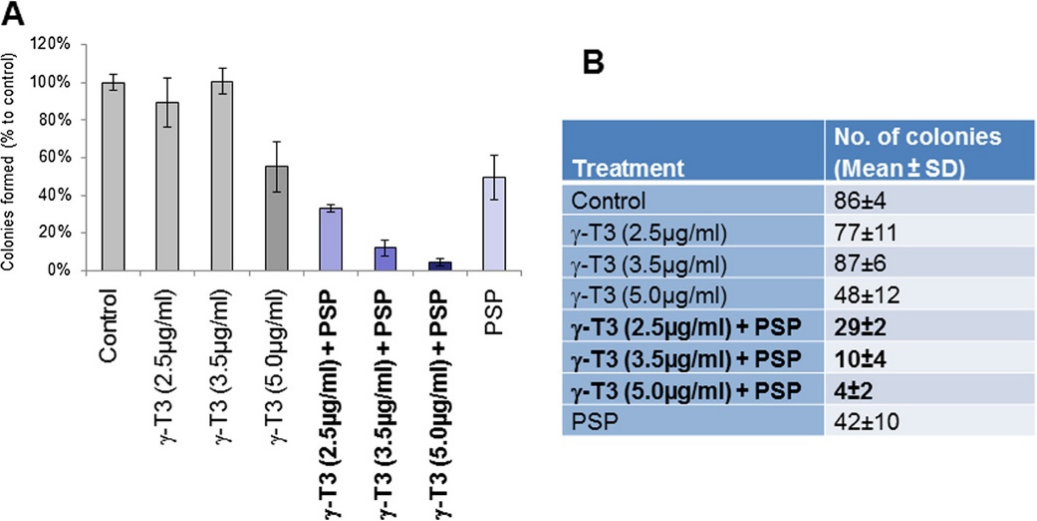
***Inhibition of colony forming ability by PSP and*** **γ-T3. (A&B)**. PC-3 cells were allowed to grow under different combinations of PSP and γ-T3. Colonies formed at the end of the experiment were stained and counted. The result was presented as percentage relative to control **(A)** or as actual colony no **(B)**. All data represent mean and SD.

To study the synergistic effect between PSP and γ**-**T3, colony formation assay was performed with prostate cancer cell line PC3 in the presence or absence of PSP and/or γ**-**T3. As shown in the Figure 1, PSP at 30 μg/ml resulted in a decrease of colonies (~50%) when compared to control. Interestingly, γ**-**T3 at concentrations of 2.5 μg/ml and 3.5 μg/ml did not have any obvious effect on colony forming ability of the cells. However, when administered simultaneously with PSP, it reduced the number of colonies by approximately 65% and 85%, respectively. In addition, 5 μg/ml of γ**-**T3 leads to a 45% reduction in cell number, which when combined with PSP, almost led to complete inhibition of colony formation. These data suggest that PSP and γ**-**T3 work synergistically in reducing the viability of the cancer cells.

### Synergistic effect of PSP and γ-T3 on AMPK activation

**Figure 2:**
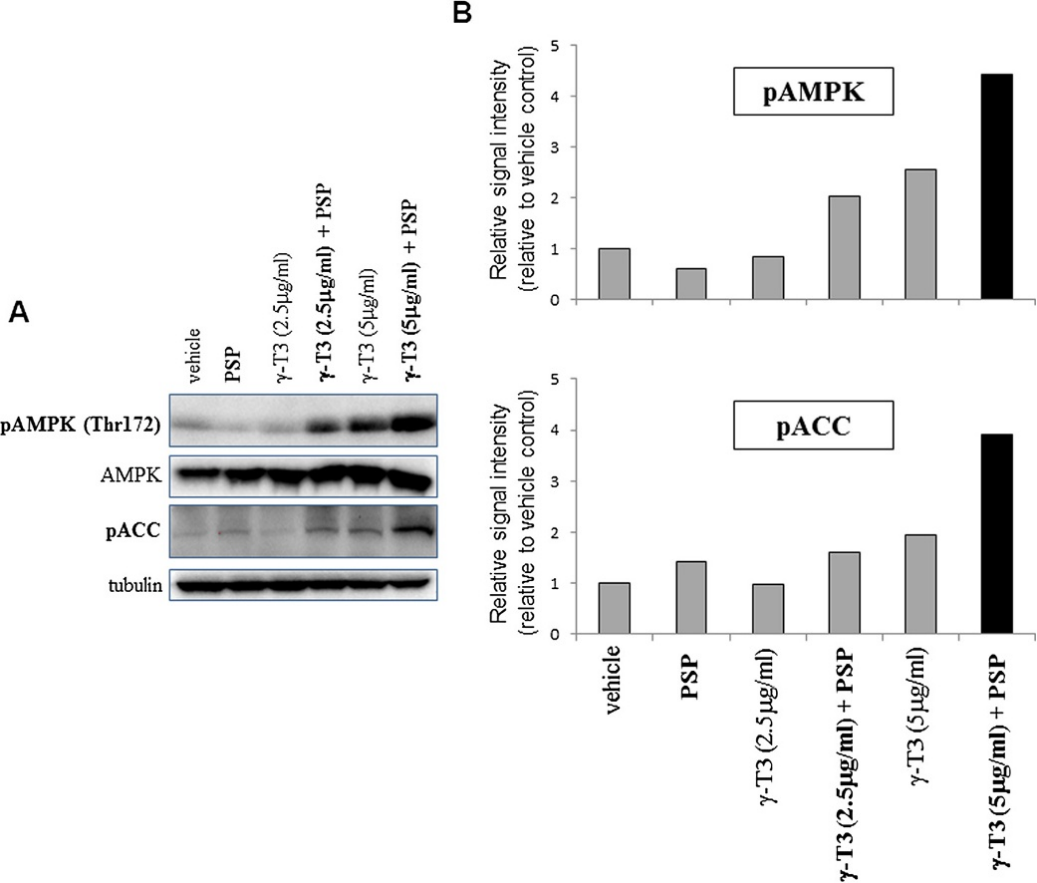
***Effect of PSP and γ-T3 treatments on AMPK signalling pathway.***PC-3 were treated with the indicated compounds for 24 hr. **(A)** Levels of phospho-AMPKα (Thr172), phospho-ACC (Ser79) and tubulin (as loading control) were examined by Western blotting. **(B)** Western blot result was quantitated with gel documentation system and the data was presented as fold change relative to the control.

To examine the potential mechanism behind the synergistic effect between PSP and γ**-**T3, we then examine the combine effect of PSP and γ**-**T3 on a series of cell signalling pathways involved in CSC survival/metabolism. While the effect of γ**-**T3 on the cell survival pathways such as AKT and JNK was not affected by PSP treatment (data not shown), we found that when the cells were treated with 2.5 μg/ml or 5 μg/ml of γ**-**T3 in combination with 30 μg/ml of PSP for 24 hr, the phosphorylation levels of AMPK at Thr172 were significantly up-regulated in the PSP and γ-T3 combination group compared with either PSP or γ-T3 (Figure 2). Meanwhile, the total AMPK protein levels were not significantly affected by the treatments, suggesting that the induction of AMPK phosphorylation is not a result of increased AMPK transcriptional level. It is notable that although γ-T3 alone at 5 μg/ml also leads to the phosphorylation of AMPK, a more profound effect was observed in the combination therapy group (Figure 2). Strikingly, the combined treatment of PSP and γ**-**T3 also upregulated the phosphorylation levels of ACC, a direct downstream target of AMPK. Since ACC phosphorylation by AMPK results in inactivation of its activity, our findings suggest that PSP and γ-T3 cooperate to promote AMPK activation, leading to suppression of the ACC function.

### Combination of PSP and γ-T3 is a novel strategy against prostate cancer

**Figure 3:**
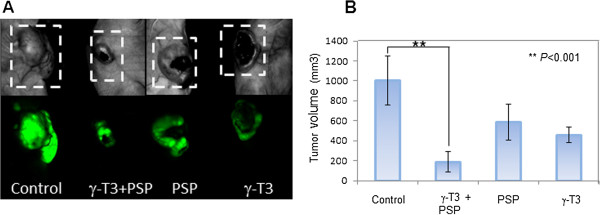
***In vivo treatment of PSP and γ-T3.***The *in vivo* therapeutic effect of PSP and γ-T3 was examined in a nude mouse model with PC3-GFP cells. Nude mice were randomized into four groups, each consisting of five animals. Each group was treated daily for 30 days with either γ-T3 (50 mg/Kg/day), PSP (300 mg/Kg/day), γ-T3 (50 mg/Kg/day) plus PSP (300 mg/Kg/day and or DMSO and PBS as the control group. **(A)** Tumor volume in each group was evaluated using Maestro imaging system. Representative mice in each group were shown. **(B)** γ-T3 administration reduced the tumor size by ~2 folds as compared with control and its effect was similar to PSP alone. The combination of γ-T3 and PSP exhibited a synergistic effect on tumor suppression (P < 0.01, *t* test).

**Figure 4:**
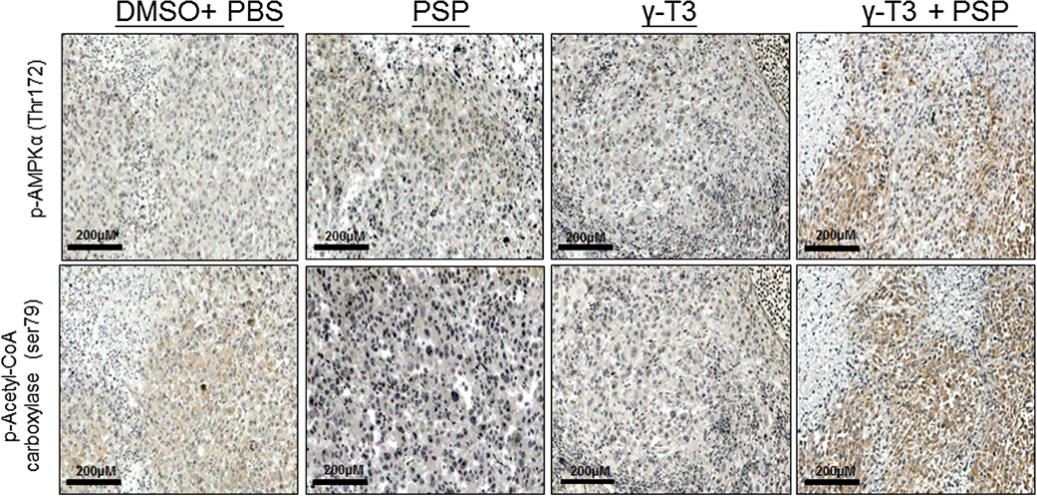
***PSP and γ-T3 activates AMPK signalling pathway in vivo.***Immunostaining was performed on xenograft tumor collected from the mice with the indicated treatment. Consecutive sections were stained with antibody against phospho-AMPKα (Thr172) and phospho-ACC (ser79). Note that an increase in cytoplasmic expression of both proteins was observed in tumors co-treated with γ-T3 and PSP (200× magnifications).

We examined the therapeutic role of combined effect of γ-T3 and PSP *in vivo* using PC3-GFP cells. Athymic nude mice were allografted with PC3-GFP cells and were divided into γ-T3 alone, PSP alone, combined γ-T3 and PSP and control (DMSO and PBS). After 30 days of treatment, the tumor was imaged using Maestro imaging system. The representative mice are shown in Figure 3A. γ-T3 reduced the tumor volumes in a manner approximately two folds and the effect is similar to PSP. In addition, γ-T3 combined with PSP had significant inhibitory effects on PC3-GFP growth *in vivo* compared to the DMSO and PBS control group (P < 0.01) (Figure 3B). During the experiment, no signs of toxicity (infection, diarrhea, or loss of body weight) were observed in the animals undergoing treatment with PSP and γ-T3 when compared with DMSO and PBS. To confirm whether the growth suppressive effect γ-T3 in combination with PSP is due to induction of autophagy, we examined and compared the expression of phospho-AMPKα (Thr172) and phospho-ACC (Ser79) in the tumor xenograft tissues of treated and control groups. Consistent to the *in vitro* observation (Figure 2), we found that increased expression in xenograft tumor of both phospho-AMPKα (Thr172) and phospho-ACC (Ser79) in mice treated with γ-T3 and PSP when compared with the control (Figure 4), suggesting that γ-T3 in combination with PSP leads to AMPK activation and subsequent inactivation of ACC *in vivo*.

## Discussion

PSP and γ-T3 have been studied extensively in separate studies for their potent anti-cancer effects against a wide-range of cancer cells which includes breast [9–11], liver [12], prostate cancer [13] and melanoma. Despite of this, the mechanisms underlying their anti-cancer effects remain poorly understood. Here, we demonstrated for the first time that PSP acts synergistically with γ-T3 to suppress the survival of prostate cancer cells. This combined effect of PSP and γ-T3 was associated with activation of the AMPK signalling pathway, leading to inactivation of the ACC. The synergistic effect of PSP and γ-T3 was further confirmed with a prostate cancer xenograft models.

γ-T3 is one of the constituents of Vitamin E which has been shown to possess anti-oxidative, anti-cardiovascular disease, neuroprotective and anticancer effects [14]. Recently, γ-T3 was also reported to sensitize cancer cells to chemotherapeutic drugs [5, 15]. γ-T3 is known to target a number of key cell signalling pathway frequently activated in cancer cells. Meanwhile, γ-T3 treatment has also been shown to induce autophagy in cancer cells. Although AMPK signalling cascade plays a key role in the induction of autophagy in normal and cancer cells [16], whether γ-T3 regulates AMPK in cancer cells is currently unclear. The fact that both phosphorylated AMPK and ACC were upregulated by γ-T3 treatment clearly support that γ-T3 activates AMPK and its downstream signalling cascade in cancer cells. Meanwhile, this effect of γ-T3 appears to be dose dependent as a lower dosage of γ-T3 (2.5 μg/ml) failed to produce any observable changes on AMPK and ACC phosphorylation.

Unlike γ-T3, PSP has not been shown to regulate either the cell survival signalling pathways or the metabolic pathway like AMPK. As expected, treatment of cancer cells with PSP was unable to induce the phosphorylation of either AMPK or ACC. It is therefore surprising that in the presence of PSP, the effect of γ-T3 was drastically enhanced. Indeed, at a dose of γ-T3 that failed to induce AMPK activation, addition of PSP was found to significantly upregulate AMPK activation. Since the majority of the research on PSP were restricted on its immunomodulatory effect, it is currently unclear how PSP enhanced the effect of γ-T3. However, considering the important role of AMPK activation in inhibiting stem cell self-renewal [16] and that both γ-T3 and PSP were found to have potent anti-CSC [2, 4], it is tempting to speculate that by combining PSP and γ-T3, a more potent anti-CSC effect can be achieved. Indeed, co-treatment of PSP and γ-T3 were found to achieve a significant tumor suppressive effect *in vivo*. Meanwhile, examination of the tumor tissues after the co-treatment confirmed that AMPK and ACC phosphorylation were both upregulated.

## Conclusion

In summary, we have demonstrated for the first time that PSP not only enhance the effect of γ-T3 on AMPK activation, but also showed that it effectively inhibits tumor growth *in vivo*. Our results suggest that combination of PSP and γ-T3 may be an effective therapeutic strategy for the treatment of cancers.

## Abbreviations

AAC: Acetyl-CoA carboxylase
AMPK: AMP-activated protein kinase
CSCs: Cancer stem cells
γ-T3: Gamma-tocotrienols
PSP: Polysaccharopeptide.

## References

[1] de Wit R (2005). Shifting paradigms in prostate cancer; docetaxel plus low-dose prednisone - finally an effective chemotherapy. Eur J Cancer.

[2] Luk SU, Yap WN, Chiu YT, Lee DT, Ma S, Lee TK, Vasireddy RS, Wong YC, Ching YP, Nelson C, Yap YL, Ling MT (2011). Gamma-tocotrienol as an effective agent in targeting prostate cancer stem cell-like population. Int J Cancer.

[3] Lee TK, Castilho A, Cheung VC, Tang KH, Ma S, Ng IO (2011). Lupeol targets liver tumor-initiating cells through phosphatase and tensin homolog modulation. Hepatology.

[4] Luk SU, Lee TK, Liu J, Lee DT, Chiu YT, Ma S, Ng IO, Wong YC, Chan FL, Ling MT (2011). Chemopreventive effect of PSP through targeting of prostate cancer stem cell-like population. PLoS One.

[5] Yap WN, Chang PN, Han HY, Lee DT, Ling MT, Wong YC, Yap YL (2008). Gamma-Tocotrienol suppresses prostate cancer cell proliferation and invasion through multiple-signalling pathways. Br J Cancer.

[6] Ahn KS, Sethi G, Krishnan K, Aggarwal BB (2007). Gamma-tocotrienol inhibits nuclear factor-kappaB signaling pathway through inhibition of receptor-interacting protein and TAK1 leading to suppression of antiapoptotic gene products and potentiation of apoptosis. J Biol Chem.

[7] Samant GV, Sylvester PW (2006). Gamma-Tocotrienol inhibits ErbB3-dependent PI3K/Akt mitogenic signalling in neoplastic mammary epithelial cells. Cell Prolif.

[8] Wong CK, Bao YX, Wong EL, Leung PC, Fung KP, Lam CW (2005). Immunomodulatory activities of Yunzhi and Danshen in post-treatment breast cancer patients. Am J Chin Med.

[9] Ho CY, Kim CF, Leung KN, Fung KP, Tse TF, Chan H, Lau CB (2005). Differential anti-tumor activity of coriolus versicolor (Yunzhi) extract through p53- and/or Bcl-2-dependent apoptotic pathway in human breast cancer cells. Cancer Biol Ther.

[10] Chow LW, Lo CS, Loo WT, Hu XC, Sham JS (2003). Polysaccharide peptide mediates apoptosis by up-regulating p21 gene and down-regulating cyclin D1 gene. Am J Chin Med.

[11] Wan JM, Sit WH, Louie JC (2008). Polysaccharopeptide enhances the anticancer activity of doxorubicin and etoposide on human breast cancer cells ZR-75-30. Int J Oncol.

[12] Dong Y, Kwan CY, Chen ZN, Yang MM (1996). Antitumor effects of a refined polysaccharide peptide fraction isolated from Coriolus versicolor: in vitro and in vivo studies. Res Commun Mol Pathol Pharmacol.

[13] Hsieh TC, Wu JM (2001). Cell growth and gene modulatory activities of Yunzhi (Windsor Wunxi) from mushroom Trametes versicolor in androgen-dependent and androgen-insensitive human prostate cancer cells. Int J Oncol.

[14] Sen CK, Khanna S, Roy S (2006). Tocotrienols: Vitamin E beyond tocopherols. Life Sci.

[15] Chang PN, Yap WN, Lee DT, Ling MT, Wong YC, Yap YL (2009). Evidence of gamma-tocotrienol as an apoptosis-inducing, invasion-suppressing, and chemotherapy drug-sensitizing agent in human melanoma cells. Nutr Cancer.

[16] Mihaylova MM, Shaw RJ (2011). The AMPK signalling pathway coordinates cell growth, autophagy and metabolism. Nat Cell Biol.

[CR17] The pre-publication history for this paper can be accessed here: http://www.biomedcentral.com/1472-6882/14/303/prepub

